# Clinical assessment of growth patterns, weight, and dietary management in children and adults with phenylketonuria – a retrospective study

**DOI:** 10.1186/s13023-025-04136-x

**Published:** 2025-12-13

**Authors:** Lionesa Bahtiri, Christine Jørgensen, Kirsten Ahring, Helle Rasmussen, Janne Meedon Hermansen, Olivia Welle Fjellbirkeland, Allan Meldgaard Lund, Mette Cathrine Ørngreen

**Affiliations:** Department of Clinical Genetics and of Pediatrics and Adolescent Medicine, Centre for Inherited Metabolic Diseases, Rigshospitalet, Copenhagen University Hospital, Blegdamsvej 9, Copenhagen, 2100 København Denmark

## Abstract

**Background:**

Phenylketonuria (PKU) is an inherited metabolic disorder where the body cannot break down phenylalanine (Phe), leading to its harmful accumulation if left untreated. Concerns have been raised about growth and risk of overweight in PKU patients, with conflicting research findings. This retrospective study aims to assess growth patterns and the prevalence of overweight and obesity in Danish children and adults with PKU by comparing them with Danish growth charts and prevalence rates.

**Results:**

Data were collected from medical records of 291 Danish patients from the National PKU Clinic at Rigshospitalet in Copenhagen, including data on age, sex, PKU phenotype, Phe levels, and anthropometric measurements from their last clinic visit. Weight status for children was classified according to Danish guidelines, while World Health Organization criteria were used for adults. All data analyses were carried out separately for children and adults. A total of 291 PKU patients were included, 116 children and 175 adults. Children had normal growth patterns compared to Danish growth charts. The rates of overweight and obesity among children were 15% and 1%, respectively, while 32% of adults were overweight, and 28% were obese. Adults with classic PKU were significantly more obese and had higher BMI levels compared to other phenotypes. Additionally, a slight positive correlation was noted between high Phe levels and the risk of being overweight.

**Conclusions:**

Children with phenylketonuria following a restricted diet achieve normal growth. However, overweight and obesity rates rise with age, particularly in adults with the most severe phenotype, suggesting disease severity may influence weight gain. The potential link between high phenylalanine levels and overweight requires further investigation. These findings highlight the need for ongoing weight and metabolic monitoring, as well as strategies to support weight management in adults with phenylketonuria.

## Introduction

Phenylketonuria (PKU) is an inherited metabolic disease caused by pathogenic variants in the Phenylalanine Hydroxylase (PAH) gene. These variants lead to a total or partial deficiency of the hepatic enzyme PAH and subsequent phenylalanine (Phe) accumulation in the body [1]. PKU is rare, with prevalence varying by ethnicity and geographic region. In Denmark 5 to 10 children are born with PKU each year [2–5].

Untreated, PKU leads to irreversible central nervous system damage. Early intervention, recommended within the first 10 days of life, is crucial for normal development. Traditionally, PKU management has relied on a Phe-restricted diet, limiting dietary protein and supplementing with Phe-free amino acid (AA) supplements and low-protein (Lp) products. Other therapeutic options in PKU include sapropterin dihydrochloride, a synthetic form of the PAH cofactor, which lowers Phe levels in patients with sufficient residual enzyme activity, and Large Neutral Amino Acids (LNAA) that reduce Phe influx at the blood-brain barrier, making a less stringent dietary protein restriction possible [1, 6]. Recently, Pegvaliase (a Phe ammonia lyase that breaks down Phe upon parenteral administration) has become available in some countries, which may remove the need for diet [7].

Despite advances in treatment, most PKU patients must adhere to a Phe-restricted diet from infancy onward. Due to the restriction of natural protein sources, these diets often result in a higher intake of fat and carbohydrates to meet energy requirements [8]. This involves replacing natural protein sources with a semisynthetic diet with Phe-free AA mixtures. However, the reduced natural protein in this diet may result in increased hunger [9]. While whole-body protein metabolism in PKU patients is similar to that in healthy controls [10], experimental and clinical studies suggest that consuming natural protein rather than an AA mix results in lower nitrogen excretion for the same energy and AA intake, indicating that AA supplements are less bioavailable [11–13]. Some authors have proposed that improving the quality of protein substitute, rather than increasing total protein intake, may be more important for physical growth [14]. However, studies on this topic have yielded contradictory findings [15–18].

The literature on physical growth in children with PKU presents inconsistent findings. Some earlier studies suggest that physical growth in children with PKU is impaired [19], while other studies indicate that normal growth can be maintained regardless of PKU severity [20]. Similarly, some studies report a tendency towards obesity in both adults and children with PKU [21–23], whereas others cannot reproduce this [24, 25]. These inconsistencies may be due to regional differences in treatment protocols, dietary management, access to care, and variations in study populations [26].

Our study aimed to provide a comprehensive assessment of growth and weight patterns in children and adults with different phenotypes and given different treatment modalities, focusing on all PKU patients followed at the national PKU clinic in Copenhagen, Denmark.

## Material and methods

### Study design

This is a single-center, retrospective study of PKU patients managed in the PKU clinic at Rigshospitalet in Copenhagen, which is the only PKU Clinic in Denmark. This clinic manages approximately 540 patients including late-diagnosed PKU patients. The study protocol was approved by Copenhagen University Hospital, Rigshospitalet, Department of Health Law (*p*-2024–15732). The approval allowed access to medical records for research purposes without the patient’s explicit consent, unless the patient had submitted a direct objection in their medical record. The access allowed data retrieval from up to five years prior to the study’s initiation date. The patients were identified with the assistance of a dietitian who participates in the care of all PKU patients in Denmark.

All eligible patients were diagnosed through the Newborn Screening Program and classified according to their mutation type, which includes the four phenotypes: mild hyperphenylalaninemia (mHPA), mild PKU, moderate PKU, and classic PKU. Due to the small number of patients with moderate PKU, we combined these with the classic PKU group because of their similarity in severity and treatment protocol.

Exclusion criteria included patients who were treated late or had a second inherited metabolic or hormonal disorder (e.g. diabetes and hypothyroidism). In the case of pregnant patients, only data prior to pregnancy and the pre-conception diet were used. If no prior or post-pregnancy data were available, these patients were entirely excluded from the study.

### Data collection

Clinical data were retrospectively collected from medical records accessed via *Epic*, the current digital medical record system in Denmark. The data was collected from the most recent clinical visit. To limit registration errors, all registrations were performed by the same person.

The primary outcome measures were growth patterns and the prevelance of overweight and obesity among PKU patients. Growth patterns were assessed using z-scores for weight, length/height, BMI, and weight-for-height in children based on Danish growth charts [27]. These z-scores were collected via *Epic.* For adults, the primary data collected included height (cm) and weight (kg), which were used to calculate BMI (kg/m^2^)

Secondary outcome measures included PKU phenotype, sex, age, overall diet type, and all plasma Phe levels within the year preceding the most recent clinical visit, which were used to calculate a mean Phe level.

#### Dietary data

Dietary data were categorized according to the typical management practices in the PKU clinic. The following diets were identified: Regular PKU diet (AA-supplements + Lp products), sapropterin diet with or without AA supplement, LNAA diet (semi-free diet), and LNAA diet with AA supplement (see also below).

For children, the dietary regimens include regular PKU diet (AA-supplements + Lp products), in which patients follow a Phe-restrictive diet supplemented with AA supplements and Lp products, or a sapropterin diet with or without an AA supplement. Both regimens aim to maintain Phe blood levels between 120 and 360 µM for ages 0–12 years, and below 600 µM for ages 12 and older.

The same dietary categories apply to adults, along with two additional options: The LNAA diet (semi-free diet) that provide more flexibility for adults by allowing less limited eating within prescribed restrictions. LNAA contributes approximately 455 kJ/day to the total energy intake. Patients are advised to follow the general Danish dietary recommendations for fat and carbohydrate intake, with a reduced intake of meat and, if necessary, supplementation with amino acid mixtures (LNAA with AA-supplement) to ensure adequate protein intake without exceeding plasma Phe concentrations of 1500 µmol/L. Adults with PKU treated with LNAA have a protein requirement of 1.1 g/kg/day. For individuals with PKU aged 14 years and older, the general protein requirement is 1.0–1.5 g/kg/day.

In the analysis, the diet types will be divided into four overall groups: no diet, regular PKU diet, sapropterin, and LNAA. However, a subgroup of sapropterin with and without AA supplement and LNAA with and without AA supplement will be used in some analyses on growth and prevalence of overweight in both children and adults.

### Classification of overweight and obesity

The Danish national guidelines [28] were applied to estimate the prevalence of overweight and obesity among PKU patients. To account for growth and development in children and adolescents, BMI Standard Deviation Scores (SDS) or percentiles on reference charts are preferable over raw BMI values. Overweight in childhood and adolescence is defined as a BMI above the 90th percentile (equivalent to a BMI-SDS above 1.28) and obesity as a BMI above the 99th percentile (equivalent to a BMI-SDS above 2.33) when compared to Danish reference charts [27]. A BMI below the 10th percentile (equivalent to a BMI-SDS below −1.28) is considered underweight. This definition was applied to patients aged 0 to 17 years. Given a lack of BMI percentile data on 5 patients, we used BMI-SDS to estimate prevalence of overweight/obesity, ensuring that most patients were included. BMI-SDS were calculated using the LMS method [29] based on a Danish reference [27].

In Denmark, the WHO definitions of overweight and obesity in adults are used as a reference [30]. Adults with a BMI between 25 and 29.9 kg/m^2^ are considered overweight, while those with a BMI of 30 kg/m^2^ or higher are considered obese. Underweight is defined as having a BMI less than 18.5 kg/m^2^. These standards were applied to patients aged 18 and above.

### Statistical analysis

Statistical analyses were performed using the statistical software R (version 4.2.0.). Statistical significance was established when *p* < 0.05.

### Descriptive analysis

Continuous variables, such as age, weight z-score, height z-score, weight-for-height z-score and BMI-SDS were expressed as mean ± standard deviation, or as median and range if data did not follow normal distribution. Weight, height and weight-for-height z-score values between −2 and +2 were considered normal. Categorial variables, such as weight classes (underweight, normal weight, overweight and obese as defined above) are described using percentages. The numbers and percentages presented were calculated using the exact number of patients for whom data were available.

#### Comparisons between groups and associations

To control for age-related differences in dietary and metabolic parameters, all statistical analyses were done separately for children and adults.

Significant differences in z-scores for all growth parameters of children were analyzed in comparison to the Danish growth charts’ mean of 0, using a One-sample *t*-test. Differences in subgroups of phenotype and diet types in both children and adults were analyzed by one-sided ANOVA. Post-hoc pairwise comparisons were performed using Tukey HSD. In addition, a Two-sample *t*-test was conducted on comparisons between sapropterin and LNAA groups; with and without AA supplements. Chi-square test was used to determine weight prevalence differences among groups. To identify significant weight differences between groups, we used adjusted residuals with Bonferroni correction. Prevalences were compared between children and adults, females and males, various phenotypes, and diet types. Given the absence of a normal distribution for Phe levels, the non-parametric Mann-Whitney U test was used to compare Phe levels between two groups. Linear regression analysis was used to examine the relationship between Phe levels, growth parameter z-scores in children, and BMI in adults. Association between blood Phe levels and likelihood of being overweight was analyzed by logistic regression.

## Results

### PKU-population

**Fig. 1:**
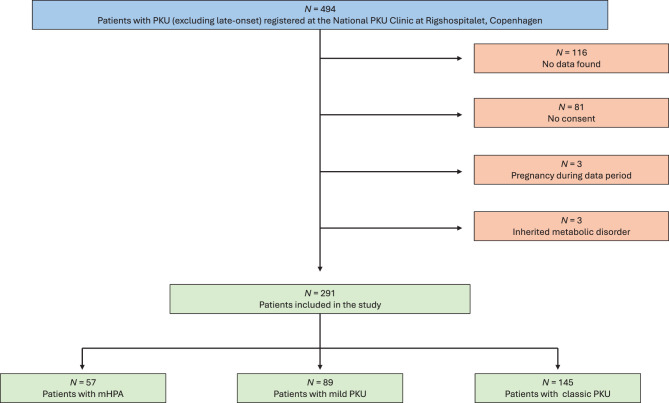
Patient flow. 291 patients with PKU were included

A total of 291 patients with PKU were included (Fig. 1). The anthropometric characteristics of the study population are summarized in Tables 1 and 2.

**Table 1:** Overview of the study population profile in age group < 18 years

	All patients (n = 116)	mHPA (n = 33)	Mild PKU (n = 35)	Classic PKU (n = 48)
**Age, Y**	8.1 ± 5.3	6.7 ± 5.7	8.8 ± 4.7	8.9 ± 5.3
**Sex**	-	-	-	-
Male	68	20	22	26
Female	48	13	13	22
**Height (z-score)**	−0.03 ± 1.03	−0.01 ± 0.95	−0.01 ± 1.20	−0.07 ± 0.97
**Weight (z-score)**	0.21 ± 1.00	0.26 ± 0.91	0.14 ± 1.16	0.22 ± 0.95
**Weight for height (z-score)**	0.36^a^ ± 1.07	0.42 ± 1.11	0.29 ± 1.05	0.36^a^ ± 1.08
**BMI (z-score)**	0.39^a^ ± 1.22	0.44 ± 1.20	0.28 ± 1.26	0.44^a^ ± 1.25
**BMI-SDS**	0.31 ± 1.1	0.33 ± 1.11	0.19 ± 1.10	0.35 ± 1.13
**Mean Phe, μmol/L**^**b**^	320 (154–1000)	252 (172–405)	323 (165–738)	364 (154 - 1000)
**No diet**	24%	79%	3%	2%
**Regular PKU diet**	40%	0%	0%	96%
**Sapropterin**	36%	21%	97%	2%

^a^*p* < 0,05 calculated by a One-sample *t*-test, compared to Danish growth charts. ^b^ Data are given as median and range

**Table 2:** Overview of the study population profile in age group ≥ 18 years

	All patients (n = 175)	mHPA (n = 24)	Mild PKU (n = 54)	Classic PKU (n = 97)
**Age, Y**	34 ± 12.1	27.5 ± 9.9	32.4 ± 9.5	38.2 ± 12.8
**Sex**	-	-	-	-
Male	75	9	26	38
Female	100	15	28	49
**Height (cm)**	171.8 ± 21.7	171.9 ± 10.2	173.8 ± 9.9	171.5 ± 9.7
**Weight (cm)**	81.8 ± 21.7	71.9 ± 18.8	82.8 ± 21.8	84.0 ±21.1
**BMI (kg/m**^**2**^)	27.7 ± 6.9	24.1 ± 4.8	27.2 ± 6.0	28.6 ± 7.2
**Mean Phe, μmol/L**^**a**^	937 (181–1850)	332 (210–1160)	499 (181–1317)	1350 (352–1850)
**No diet**	21%	92%	18%	5%
**Regular PKU diet**	11%	0%	6%	17%
**Sapropterin**	19%	4%	57%	1%
**LNAA**	49%	4%	19%	77%

^a^Data are given as median and range

Out of 291, 116 were children in the age range 0–17 years, and 175 were adults aged 18 years and above. The phenotypic distribution was as follows: 20% (*n* = 57) had a mHPA phenotype, 31% (*n* = 89) had a mild PKU phenotype and 50% (*n* = 145) had a classic phenotype. Twenty-two percent of patients followed a regular PKU diet, 30% received LNAA treatment, 26% were treated with sapropterin while the last 22% were not on any dietary restrictions or treatments.

### Anthropometric characteristics

#### Age < 18 years

**Fig. 2:**
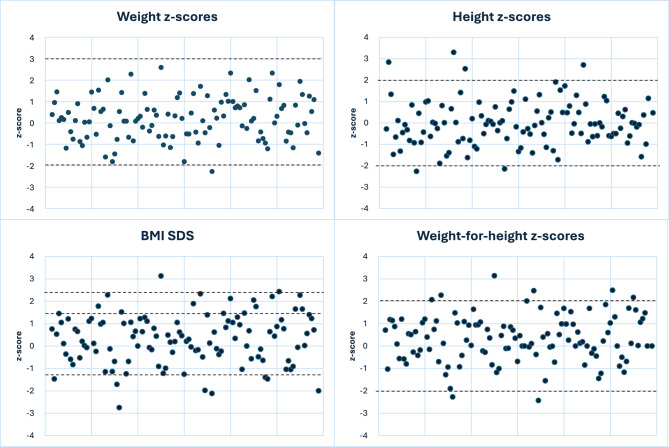
Distribution of z-scores for weight, height, and weight-for-height, and BMI-SDS for PKU children. The lines at z-scores −2 and 2, as well as BMI-SDS values −1.28 and 1.28, represent the normal range. Obesity is indicated by the 2.33 BMI-SDS-line

The anthropometric characteristics of children shown in Table 1 included height, weight, weight-for-height, and BMI z-scores. Figure 2 depicts the distribution of z-scores and BMI-SDS among PKU children. The distribution of z-scores revealed variations in growth patterns among PKU children. However, the majority of observations fell within the normal range, as shown by the lines at z-scores −2 and 2.

Weight-for-height (*p* = 0.0007) and BMI z-scores (*p* = 0.0009) were higher in PKU children compared to Danish growth charts. There was no significant difference in weight and height z-scores. When stratified by phenotypic classification, the classic phenotype demonstrated higher weight-for-height (*p* = 0.02897) and BMI z-scores (*p* = 0.02204). Comparing z-scores between phenotypes, showed no significant differences between any of the growth parameters.

When further stratified by diet type, the group on a regular PKU diet demonstrated a difference in weight-for-height (0.40 ± 1.00, *p* = 0.01062) and BMI z-scores (0.48 ± 1.15, *p* = 0.00703) when compared to Danish growth charts. A comparison of two sapropterin groups (with and without AA) revealed no significant difference in any growth parameters. No significant difference was observed between boys and girls in weight, weight-for-height, or BMI z-scores. However, height z-scores for PKU boys were higher (0.31 ± 1.08 vs. −0.29 ± 0.89 in girls, *p* = 0.01166).

#### Age$\ge$18 years

We found a significant difference in BMI levels in adults across three phenotypic groups (*p* = 0.0105). Patients with the classic phenotype had higher BMI levels than those with the mHPA phenotype (*p* = 0.0083). There was no significant difference in BMI levels between patients with the mild phenotype and those with the mHPA phenotype (*p* = 0.1465), nor between patients with the mild phenotype and those with the classic phenotype (*p* = 0.3843). No significant variations were found in BMI levels when stratified by diet types.

### Prevalence of overweight and obesity

**Fig. 3:**
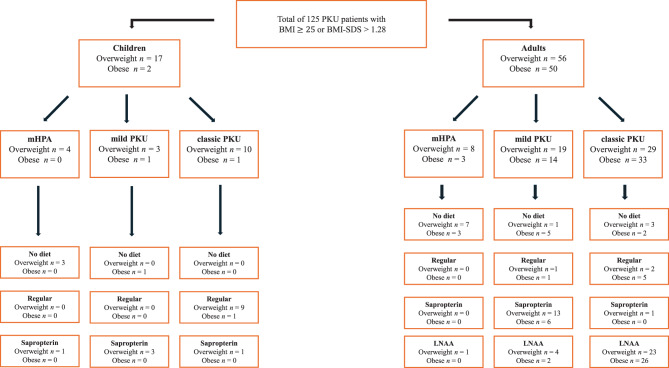
Flow diagram of all overweight and obese PKU patients

Among 116 children, 77% (*n* = 88) were normal weight, while 16% (*n* = 19) had a BMI-SDS > 1.28 with 15% (*n* = 17) being overweight, and one percent (*n* = 2) obese. Seven percent (*n* = 8) were underweight. Out of 175 adults, 37% (*n* = 64) were normal weight, and 60% (*n* = 106) had a BMI ≥ 25 with 32%(*n* = 57) being overweight and 28% (*n* = 49) obese. Three percent (*n* = 6) were underweight. The distribution of overweight and obese PKU patients is presented in a flow diagram, Fig. 3.

We found a significant difference between the prevalence of overweight (15% vs. 32%, *p* = 0.00181) and obesity (1% vs. 28%, *p* = 1.22 × 10^−8^) in children versus adults. Table 3 shows the prevalences in children and adults compared to the Danish population [31, 32]. The prevalence of overweight is presented in the same way as the data obtained from the Danish population.

**Table 3:** Prevalence of overweight and obesity in PKU patients compared to the Danish population

	PKU population	Danish population
** < 18 years**		
BMI-SDS > 1.28	16%	~13–18 [32]
BMI-SDS > 2.33	1%	~3–4 [32]
**≥ 18 years**		
BMI ≥ 25	60 %	52,6 % [31]
BMI ≥ 30	28 %	18,5 % [31]

**Fig. 4:**
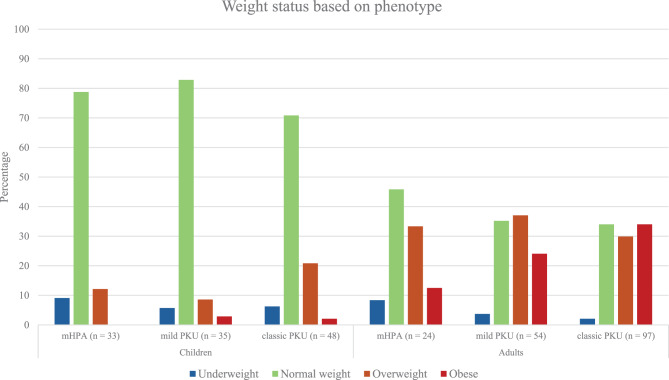
Weight status based on phenotype, classified using Danish national guidelines for participants under 18 and who definitions for those 18 and above

**Fig. 5:**
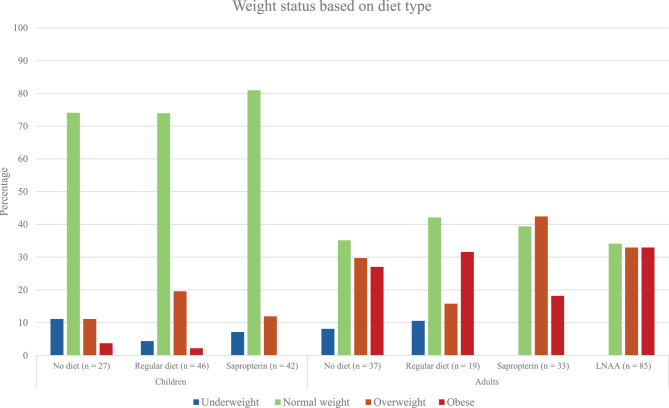
Weight status based on diet type, classified using Danish national guidelines for participants under 18 and who definitions for those 18 and above

There was no significant difference in overweight and obesity between boys and girls in children, or males and females in adults. Figures 4 and 5 presents the distribution of weight status between phenotypes and diet types in both children and adults. Separate analyses in children and adults revealed no significant difference in the prevalence of overweight among any phenotypes or diet types.

In children, no significant difference in the prevalence of obesity between phenotypes and diet types were found. However, there was a difference in obesity prevalence in adults, with results showing that classic PKU with a prevalence of 30% had a higher prevalence than both mHPA (12,5%, *p* = 0.019) and mild PKU (24%, *p* = 3.8 × 10^−8^). No difference was found in obesity prevalence between diet types in adults (*p* = 0.4476).

The groups treated with sapropterin and LNAA were further divided into those that received AA supplements and those that did not. In children, there was no difference in the prevalence of overweight (including obesity patients) between the sapropterin with AA and the sapropterin without AA (14.8% vs. 6.7%, *p* = 0.7763). Similarly, no difference was observed in the prevalence of overweight in adults between those on sapropterin with AA and without AA (50% vs. 65.2%, *p* = 0.6639) or LNAA with AA and without AA (66% vs. 64.7%, *p* = 1.000).

### Phe levels among different groups

The median Phe levels for the various phenotypes are presented in Tables 1 and 2.

Not all patients measure Phe levels regularly; thus, data were unavailable for 62 patients. Similarly, the frequency of measurements differed, and means were calculated from measurements ranging from two to one hundred.

Phe levels did not differ significantly between boys and girls in children (*p* = 0.9626) or between males and females in adults (*p =* 0.215).

### Associations of Phe levels

A logistic regression analysis on children indicated that higher Phe levels were associated with an increased likelihood of being overweight (OR = 1.004, 95% CI: 1.001–1.008, *p* = 0.022). In adults, the analysis also revealed a statistically significant, though very small, association between Phe levels and the likelihood of being overweight (OR = 1.0009, 95% CI: 1.0001–1.0017, *p* = 0.030). While significant, the effect size is negligible.

Linear regression showed that there was a significant positive association between Phe levels and BMI in adults (*p* = 0.0135). There was not a significant association between Phe levels and z-scores for height, weight, weight-for-height, and BMI in children.

## Discussion

As our knowledge of PKU increases, attention has turned to the multifaceted relationship between growth and weight management. The purpose of this study was to investigate the growth of PKU children, the prevalence of overweight and obesity among both children and adults with PKU, and the potential associations between phenotype, diet type, and Phe levels with these growth outcomes.

Our main conclusions were:

1. Danish children with PKU have normal growth.
2. Overweight and obesity prevalence in children with PKU was comparable with those in the general population.
3. Overweight and obesity prevalences in adults were higher than those in the general population.
4. In both children and adults, there was no difference in overweight prevalence across phenotypes or diet types. Adults with classic PKU, on the other hand, are more likely to be obese than other phenotypes, and their BMI levels are higher than those with mHPA.
5. Elevated Phe levels may be associated with increased risk of being overweight.

Our findings show that the z-scores for weight, length and weight-for-length in PKU children are mostly within normal ranges, indicating normal growth. Although there were significant differences in weight-for-height and BMI z-scores compared to Danish growth charts, the means do not suggest higher rates of overweight. We found no significant differences in growth parameters or overweight prevalence between the mHPA, mild, and classic PKU phenotypes. Overall, our findings demonstrate normal growth patterns in PKU children, with no increased prevalence of overweight or obesity compared to the Danish population. A systematic review found overweight rates in PKU children and adolescents ranging from 8% to 33% [33], while our study found a prevalence of 16%, placing our population in the middle range of these previous findings.

Ensuring optimal growth while maintaining safe Phe levels is crucial for PKU patients. Although some studies associate high protein intake in infancy with childhood overweight and obesity [34, 35], our PKU children do not tend to be overweight despite receiving 3 grams of protein per kilogram of body weight from birth gradually decreasing to 2 gram per kilogram during first year. Other studies specifically link excessive protein intake from animal foods in infancy to obesity [36, 37]. However, the literature on this association remains inconsistent [34].

Though our patients consume a large part of their protein from AA supplements recent research on PKU has shifted the focus from total protein intake to the source of protein, suggesting that a natural source is more beneficial for growth than synthetic substitutes [14]. While our study did not find impaired growth as other studies have [19], we attempted to investigate the impact of synthetic AAs versus natural protein on growth in children treated with sapropterin but observed no significant differences.

While children with PKU exhibited normal growth, adults with PKU were slightly more overweight and considerably more obese than the general population. A recent systematic review reported prevalences ranging from 5% to 42% for overweight and 5% to 72% for obesity in PKU adults [38]. In our study, the prevalences were 32% for overweight and 27% for obesity. We also noted a significant difference between children and adults, suggesting an upward trend in overweight prevalence with age. This aligns with other studies indicating that PKU patients tend to gain excessive weight during adolescence [39–41], highlighting a critical period between childhood and adulthood where behavioral changes, such as non-compliance with treatment, may impact weight gain [42, 43].

PKU treatment requires strict dietary monitoring, which can lead to disordered eating and social isolation [44–46]. Adults with PKU may overindulge in permissible food due to protein restrictions. Their diet, high in fat and carbohydrates but low in natural protein, may increase hunger and overconsumption [9]. The exact factors behind the high prevalence of overweight is still unclear. Our study found a higher prevalence of obesity in adults with the classic PKU phenotype compared to other phenotypes. These patients also had higher BMI levels than those with the mHPA phenotype, aligning with previous studies showing higher BMI in classic PKU patients compared to healthy controls [47]. It is worth noting that in our study, the comparison was to mHPA, but these patients are often unaffected by the disease and can avoid treatment.

Furthermore, among 33 obese classic PKU adults, 26 followed the LNAA diet. It remains unclear whether the obesity is due to the phenotype or diet. However, there was no significant difference in overweight prevalence or BMI levels between different diet types in adults. In Denmark, LNAA treatment is offered to all patients over 18 years of age, if they request this change themselves to limit restrictions [48]; thus, this group is probably historically characterized by non-adherence and is after beginning LNAA treatment allowed Phe levels up to 1500 µM.

Lastly, our analysis found an association between mean Phe levels and the likelihood of being overweight, supporting previous studies [23, 25, 49]. However, the practical significance is limited due to the small odds ratio. We also discovered a significant relationship between Phe levels and BMI in adults. Notably, 56 of the 105 overweight adults were on an LNAA diet, which may skew results due to the high accepted Phe level at 1500 µM. These findings could suggest a possible link between metabolic control and overweight risk, but further research is needed to confirm this, considering potential confounders.

It remains unclear if current dietary and pharmacological treatments in adulthood contribute to overweight and obesity, or if early dietary treatment leads to behaviors that increase weight risk.

This study did not account for important potential confounding variables, including socioeconomic status, physical activity, psychosocial factors, family history, parental weight, or epigenetic influences, as these data were not available; these factors are, however, important determinants of BMI and growth outcomes. While BMI was used, it has limitations in assessing adiposity and body composition. Using methods such as DEXA could provide more accurate assessments and give additional context for understanding obesity in this population.

While we have classified patients according to general diet types, and a further limitation of this study is the lack of detailed information on both total caloric intake and protein consumption, which restricts our ability to fully evaluate the relationship between dietary intake and growth or weight outcomes.

Lastly, the study’s generalizability may be limited due to its focus on the Danish population, its conduct at a single national center, and the use of Danish growth charts in children. This should be taken into account when interpreting the findings, and future studies in larger, more diverse populations will be important to determine whether the results can be applied more broadly. The cross-sectional design of this study also limits its ability to provide insight into longitudinal weight gain. In particular, longitudinal research is needed to better understand the impact of dietary treatments on growth and weight, and to refine dietary guidelines for improved health outcomes.

## Conclusion

In conclusion, our study highlights that growth is not impaired in children on a Phe-restricted diet with protein intake exceeding the recommended daily allowance. Additionally, children with PKU are no more overweight than their peers. However, adults show slightly higher rates of overweight and substantially higher rates of obesity compared to the Danish population, indicating an upward trend in overweight prevalence with age. Specifically, adults with classic PKU have notably higher obesity rates and BMI levels compared to other phenotypes, suggesting that the severity of PKU may influence the risk of becoming overweight. The increased incidence of overweight and obesity may be related to high Phe levels, but this association and the underlying causes have yet to be investigated further. These findings emphasize the importance of carefully monitoring weight and metabolic health in people with PKU, especially in adulthood. Nonetheless, additional research is needed to investigate the long-term implications of these findings and develop strategies to help adults with PKU manage their weight more effectively.

## Data Availability

The datasets used and/or analysed during the current study are available from the corresponding author on reasonable request.
